# Curriculum Innovation: The Osler Apprenticeship in Neurology: Building a Sustainable Pathway for Learners Interested in Medical Education

**DOI:** 10.1212/NE9.0000000000200218

**Published:** 2025-06-10

**Authors:** Rebecca E. Khamishon, Grant Wilson, Julie Yi, Sophie Vo Huynh, Charlene E. Gamaldo, Doris G. Leung, Sean Tackett, Roy E. Strowd, Amit Pahwa, W. Christopher Golden, Carlos G. Romo, Rachel Marie E. Salas

**Affiliations:** 1 Department of Neurology, Johns Hopkins University, Baltimore, MD;; 2 Department of Medicine, Johns Hopkins University, Baltimore, MD;; 3 University of Texas at Dallas;; 4 Neuromuscular Division, Department of Neurology, Johns Hopkins University, Baltimore, MD;; 5 Division of General Internal Medicine, Johns Hopkins University, Baltimore, MD;; 6 Eudowood Neonatal Pulmonary Division, Department of Pediatrics, Johns Hopkins University, Baltimore, MD; 7 Departments of Pediatrics and Medicine, Johns Hopkins University, Baltimore, MD; and; 8 Division of Neonatology, Department of Pediatrics, Johns Hopkins University, Baltimore, MD.

## Abstract

**Introduction and Problem Statement:**

Few formal programs provide structured training in medical education (MedEd) early in a learner's career. MedEd training curricula for medical students may cultivate student understanding (and thus interest) in a MedEd career. The Johns Hopkins Osler Apprenticeship (OA) in Neurology is a structured 1-year program for senior medical students (Osler Apprentices/OAs) created to develop talent in MedEd.

**Objectives:**

By the end of the program, OAs will be better able to (1) cultivate and sustain longitudinal mentorship relationships; (2) design, implement, and present a MedEd project; (3) understand individual leadership strengths; (4) develop and refine skills in educational leadership and integrate feedback to enhance learning experiences; and (5) develop an understanding of the responsibilities of an academic educator.

**Methods and Curriculum Description:**

The OA is a 1-year program that provides learners with comprehensive exposure to MedEd through key program components of mentorship, a scholarly project, strength coaching, and leadership and experiential opportunities. To evaluate the program's impact on OAs and preceptor motivations for engagement and its benefits, we administered 3 distinct surveys to OAs at exit, OA alumni, and faculty preceptors. We implemented the 4 levels of the New World Kirkpatrick Model to evaluate the impact of the program.

**Results and Assessment Data:**

Twenty-four of 33 OAs (73%), 18 of 29 alumni (62%), and 6 of 7 preceptors (86%) responded to the surveys. Twenty-one OAs (88%) and 18 alumni (100%) recommended the OA to medical students (level 1). Alumni felt confident participating in educational activities (n = 15/83%), research (n = 15/83%), and leadership (n = 12/67%). Thirty-one OAs (94%) produced educational scholarship (level 2). OAs (n = 22/92%) and alumni (n = 14/78%) agreed that the OA influenced their academic practices and behaviors, and 7 alumni (39%) were recognized or awarded (level 3). Ten alumni (56%) have an educational role (level 4). Preceptors had scholarly output from the OA and reported career or personal development as key factors for participation.

**Discussion and Lessons Learned:**

The OA made effective progress toward building a pathway for medical educators while supporting the careers of faculty, allowing for program sustainability. These findings suggest the OA may serve as a prototype for more formalized training programs in neurology MedEd.

## Introduction and Problem Statement

The development of neurology educators has progressed through the delineation of teaching roles, implementation of specific education promotion tracks, and creation of pathways to obtain medical education (MedEd) expertise. However, exposure to the key roles of the medical educator including leadership, mentorship, teaching, and scholarship is limited.^1-4^ While postgraduate MedEd training opportunities in neurology have expanded, early introduction to the MedEd pathway may cultivate student understanding (and thus interest) in a MedEd career.^3,5,6^ Participation in research or exposure to an inspirational academic role model or mentor has been demonstrated to increase student interest in academic medicine careers.^5,7-11^ Initiatives such as the American Academy of Neurology (AAN) Student Interest Group in Neurology have contributed to informal MedEd mentoring within neurology clerkships nationwide. However, few standardized MedEd training programs exist for medical students both within and outside neurology, with most primarily focusing on research rather than providing a comprehensive and longitudinal overview of the MedEd professional pathway.^12-17^

Early mentorship in MedEd has influence on personal development and career choice and can result in increased scholarly productivity, faster academic promotion, greater faculty retention, and career satisfaction.^18-21^ However, medical educators have reported difficulty recruiting faculty for teaching and mentorship because many are not compensated for their time and service.^22-24^ These challenges pose a threat to faculty vitality, particularly in neurology, where burnout rates are high and work-life balance satisfaction is low in comparison with other specialties.^25-27^ Understanding faculty motivation and the outcomes of participation can offer insight into MedEd program sustainability and preceptor retention.^28,29^

The Johns Hopkins Osler Apprenticeship (OA) in Neurology is a structured 1-year program initiated as a means to develop talent in MedEd. The OA exposes senior medical students to the skills central to pursuing a MedEd career while also providing a platform for faculty mentorship and productivity. In this study, we describe the OA in Neurology. Using the 4 levels of the New World Kirkpatrick Model (NWKM), we conduct an evaluation of the OA to determine its impact and whether it serves as a pathway to a career in MedEd. We also examine preceptor motivations for engagement and benefits of participation in the OA to gain insight into program sustainability.

## Objectives

### Program Objectives/Goals

1. To provide senior medical students the opportunity to gain experience and exposure to the skills central to pursuing a clinician-educator academic pathway.
2. To execute an effective program that promotes excitement about and facilitates innovation in neurology in both learners and faculty educators, serving as a “win-win.”
3. To cultivate an educational environment that promotes scholarship, recognition, and engagement in pedagogical methods.

### Learner Objectives/Goals

By the end of the program, through key components (listed as follows), medical students, known as Osler Apprentices (OAs), will be better able to:

1. Cultivate and sustain longitudinal mentorship relationships.
2. Design, implement, and present a MedEd project.
3. Understand individual leadership strengths.
4. Develop and refine skills in educational leadership and integrate feedback to enhance learning experiences.
5. Develop an understanding of the responsibilities of an academic educator.

## Methods and Curriculum Description

### Development and Execution

The OA is modeled after the “PreDoc Program,” a longitudinal apprenticeship started in 2008 at Johns Hopkins University (JHU) as an undergraduate credit-based elective to encourage careers in academic medicine.^30^ Seeking to cultivate future medical educators, its founder (Neurology Core Clerkship [NCC] Leadership Faculty R.M.E.S.) expanded the model to medical students, focusing on neurology MedEd and integration with the NCC. The OA seeks to enhance medical students' exposure to key roles of a medical educator while creating an experience that inspires neurology faculty engagement. Leadership and change agency, core domains of Health Systems Science (HSS), are foundational principles of the program.

The OA was implemented in 2011 in the Department of Neurology by NCC co-directors (R.M.E.S. and C.E.G.). It was created as a MedEd research elective, allowing students to earn official credit for participating. Students could work activities into their schedule, rather than having a short, contiguous window of dedicated elective time. There was no full-time equivalent support for the directors, so preceptor involvement was initially limited to co-directors and the productivity and mutual benefits for preceptors and OAs served as the primary fuel to maintain the program. Faculty members who had previously expressed interest in MedEd initiatives within the NCC were recruited over time and matched with an OA project aligned with their expertise. In recognition of their contributions, they were given the title of “Osler Preceptors in Neurology.”^31^

### Eligibility and Selection Process

Because OAs are involved in leading the NCC, third-year medical students who complete the clerkship with high pass or honors grades are eligible to apply for the program and invited to submit online applications. The application process includes a statement of interest, curriculum vitae (CV), and transcript, followed by an interview with the co-directors. There are 1–10 acceptances per year (average 4–6).

### Key Program Components

**Figure 1:**
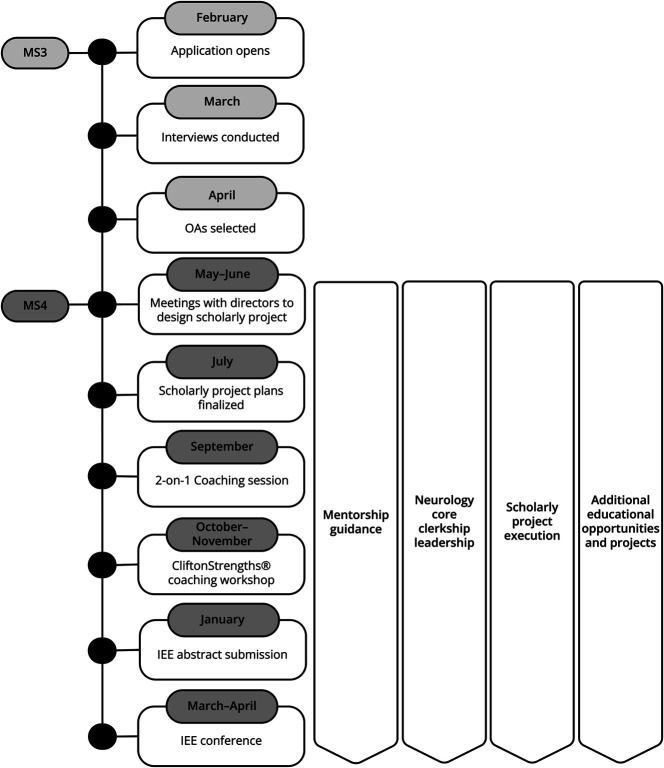
Program Timeline Abbreviations: MS3- Medical student 3rd year; MS- Medical student 4th year; OAs- Osler Apprentices; IEE- Institute for Excellence in Education.

The program time line, highlighting key program components (detailed further), is illustrated in Figure 1. The OA is designed with key foundational components (mentorship guidance, scholarly project, strengths coaching, NCC leadership) while intentionally maintaining flexibility to accommodate each Osler Apprentice's schedule and individual interests. OAs are responsible for scheduling meetings and advancing project milestones to gain hands-on experience in effectively organizing and leading initiatives within MedEd. The program remains dynamic, evolving in response to institutional and global circumstances, which allows for new projects and educational/leadership opportunities each year.

#### Mentorship Guidance

At the start of the program, OAs discuss interests and goals with the co-directors. Co-directors guide OAs to select a scholarly project, with most of the projects under their mentorship, incorporating additional faculty support as needed. OAs continue to attend monthly one-on-one meetings with directors as a means to provide a formal longitudinal apprenticeship with an educator, follow up on scholarly projects, and provide feedback.

#### Scholarly Project

Students select a scholarly project to be designed and executed during the 1-year apprenticeship with the directors' guidance and approval. To promote engagement from the preceptor, learner, and NCC, projects focused on improving Neurology Department or Clerkship productivity and/or quality are encouraged. Projects typically focus on curriculum innovation within the NCC, HSS (e.g., patient safety and quality improvement in education), health humanities, and interprofessional education. It is an expectation that a scholarly product (i.e., poster, abstract, presentation, or manuscript) will be produced and presented at either the JHU School of Medicine (SOM) Institute for Excellence in Education (IEE) Conference or another local or national conference (Table 1). Co-directors play a crucial role in guiding OAs through project feasibility within the available time frame, identifying the need for co-mentors and anticipating challenges. OAs can also build on previous projects. Undergraduate students from the PreDoc program are also recruited to project teams where they play a supportive role in research and organizational activities, which reinforces building a MedEd pipeline.

**Table 1:** Examples of OA Scholarly Projects During Academic Years 2022–2023 and 2023–2024 and Additional Published Projects

Title	Scholarly output
Education Research: Introduction of a Standardized Communication Card to Facilitate Patient-as-Teacher Training for Medical Students in the Neurology Clerkship	Manuscript: Priego-Pérez C, Arpornsuksant P, Salas RME, et al. Education Research: Introduction of a Standardized Communication Card to Facilitate Patient-as-Teacher Training for Medical Students in the Neurology Clerkship. *Neurol Educ*. 2024;3(1):e200115
Teaching Neuroethics Through a Case-Based Undergraduate Medical Education Workshop	Manuscript: Koretzky MO, Burapachaisri K, Clark B, et al. Curriculum Innovation: Teaching Neuroethics Through a Case-Based Undergraduate Medical Education Workshop. *Neurol Educ*. 2023;2(2):e200070 Abstract at IEE conference
Looking Backwards to Look Forward: An Analysis of Clinical Reasoning Reports in the Neurology Core Clerkship before and after the transition to Pass/Fail Grading	Poster at AMA Research Challenge 2023
Medical Students' Impressions of the Utility and Quality of Online Learning Modules (“Rx Bricks”) for the Neurology Core Clerkship	Presentation at IEE conference
A Strength-Based Coaching Workshop to Promote Interprofessional Collaborative Practice (IPCP) and Healthcare Professional Wellbeing	Presentation at IEE conference
The Johns Hopkins Osler Apprenticeship in Neurology: A formal evaluation of a model for medical student training in medical education	Presentation at IEE conference
Optimizing the Flipped Classroom: Utility of Clinically Focused Online Learning Modules (“RX Bricks”) for the Neurology Clerkship	Abstract at IEE conference
How a Physician is Made: Perspectives of Physical Exam Teaching Associates in the Neurology Core Clerkship	Abstract at IEE conference
Expansion of online clinical reasoning skills development in the Neurology Core Clerkship	Abstract at IEE conference
Post-COVID-19 Trends in Medical Student Procedural Exposure in the Neurology Clerkship	Abstract at IEE conference

Additional projects	Reference
American Medical Association InsideTheBoards podcast	InsideTheBoards: Health Systems Science. insidetheboards.com/Health-Systems-Science/
Virtual Elective in Equitable Healthcare	Gummerson CE, Lo BD, Porosnicu Rodriguez KA, et al. Broadening learning communities during COVID-19: developing a curricular framework for telemedicine education in neurology. *BMC Med Educ*. 2021;21(1):549
Evaluation of student impact on clinic preceptors	Tanner JA, Rao KT, Salas RE, et al. Incorporating students into clinic may be associated with both improved clinical productivity and educational value. *Neurol Clin Pract*. 2017;7(6):474-482
EMR workflow development	Tanner JA, Rao KT, Salas RE, et al. Incorporating students into clinic may be associated with both improved clinical productivity and educational value. *Neurol Clin Pract*. 2017;7(6):474-482
Review article on insomnia	Porosnicu Rodriguez KA, Salas RME, Schneider L. Insomnia: Personalized Diagnosis and Treatment Options. *Neurol Clin*. 2023;41(1):1-19

Abbreviations: EMR = electronic medical record; IEE = Institute for Excellence in Education.

#### Strengths Coaching

To aid in professional development, OAs complete a CliftonStrengths online inventory and synchronous workshop, which helps them to identify strengths and form a professional identity within MedEd. Until 2022, students underwent a strengths-focused, 1-hour, 2-on-1 coaching session with both neurology clerkship co-directors who are certified strength and master professional life coaches.

#### NCC Leadership

OAs serve as peer advisors for NCC students, acting as mentors and a bridge between clerkship students and faculty. They attend monthly administrative NCC meetings and receive deidentified clerkship feedback, providing them with experiential learning opportunities. Their responsibilities include leading monthly NCC “Tips and Tricks” workshops and neurology shelf examination review sessions and offering one-on-one peer advising sessions.^32^ They have the flexibility to design and structure sessions themselves by creating new content, incorporating preceptor feedback, or refining materials developed by previous OAs. OAs receive formative feedback from OA directors and student evaluations of their sessions.

#### Dynamic Educational Opportunities and Projects

OAs have participated in various educational opportunities and projects based on clerkship needs (Table 1). For example, during the coronavirus disease 2019 pandemic, OAs and directors developed a 2-week virtual elective on telemedicine and collaborated with undergraduate and graduate MedEd to offer a visiting elective on equitable health care. OAs have also attended a teaching assistant orientation at JHU and completed IEE self-paced courses.^33^ Co-directors support OAs in applying for awards (e.g., AAN Medical Student Prize for Excellence), and faculty may invite OAs to co-author book chapters or review articles.

### Study Design

Medical students who were selected as OAs in Neurology from 2012 through 2021 and faculty members who have served (or currently serve) as preceptors in both the OA in Neurology and OA in Pediatrics were invited to participate in surveys. Preceptors from the OA in Pediatrics (the second OA program established at Hopkins) were included to gather additional faculty/preceptor perspectives. Students who completed the program but had not yet graduated were excluded from the alumni survey. We administered 3 online, internally developed surveys (outlined further). Invitations were sent through e-mail requesting participation through respective survey platforms. A visual of survey breakdown is presented in eFigure 1 and complete surveys in eAppendixes 1–3.

1. Exit survey: administered to Neurology OAs through New Innovations software at the conclusion of their respective academic year (2012–2021). It served as a summative evaluation of the program consisting of multiple-choice, free-response, and Likert scale items. It was originally developed as a tool for formative feedback, with its questions designed to address key areas for program improvement.
2. Alumni survey: administered in 2022 to graduates/alumni of the Neurology OA program (now house staff and faculty at various institutions) through Qualtrics. It consisted of multiple-choice, free-response, and Likert scale items and an option to upload a CV. Survey items were drawn from a literature search and included adapted questions pertaining to MedEd scholarship, leadership, and professional development.^28,34^ A group of experts (R.M.E.S., C.E.G.) discussed all items. Representative participants (residents who had graduated from the OA in Pediatrics and were not part of this study) were cognitively interviewed about the survey. We made revisions based on feedback.
3. Preceptor survey: administered in 2022 to previous and current OA preceptors through Qualtrics. It consisted of multiple-choice, free-response, and Likert scale items and an option to upload a CV. To optimize content validity, we adapted items from surveys administered in previous studies.^28,35^ We discussed all items with co-authors and local educators and made revisions based on feedback.

#### Evaluation Using the NWKM

To evaluate the OA in Neurology, we used the New World Kirkpatrick Model, which expands on Kirkpatrick's original 4 levels to better embrace the complexity of educational programs and the modernization of the learning landscape.^36-39^ Level 1, “Reaction,” assesses trainee satisfaction and degree to which they find the program engaging and relevant. Level 2, “Learning,” assesses the trainee's degree of acquired knowledge, skills, attitude, confidence, and commitment. Level 3, “Behavior,” assesses whether the trainee has applied what they learned and whether they are continuing “on-the-job-learning” and includes motivators (required drivers) to maintain critical behaviors (in our case, continued involvement in MedEd) to achieve level 4. Level 4, “Results,” is the degree to which the desired outcome or organizational mission is fulfilled as a result of the training. This can be evaluated through “leading indicators,” or short-term observations and measurements (such as OA MedEd scholarly productivity or alumni continued involvement in MedEd).^36,39^

### Standard Protocol Approvals, Registrations, and Participant Consents

This study was exempted from convened review by the Johns Hopkins Institutional Review Board (IRB00307364).

### Statistical Methods

#### Quantitative and Qualitative Content Analysis

For all surveys, responses to multiple-choice and Likert-style questions were expressed as percentages. For the exit survey, qualitative content analysis with an inductive coding approach was used to analyze responses regarding program strengths and areas for improvement. Two reviewers (G.W. and S.H.) identified representative quotations through discussion. For free responses to the alumni and preceptor surveys, 2 study team members (G.W. and S.H.) performed a content analysis by independently identifying general themes and representative responses and reaching consensus through discussion. For preceptor CVs, 1 reviewer (G.W.) extracted MedEd-related scholarship activities involving both OA preceptors and alumni. Another reviewer (S.H.) searched preceptor names in PubMed between 2011 and 2023 and cross-referenced the results with names of OAs to identify MedEd-related publications. Two study team members (G.W. and S.H.) independently reviewed submitted alumni CVs and recorded MedEd activities, roles, awards, and scholarship. They resolved discrepancies through discussion.

### Data Availability

The data that support the findings of this study are available on request from the corresponding author, R.K.

## Results and Assessment Data

Since 2012, 51 medical students have enrolled in the OA program. As of 2024, 49 students have graduated with 49% (24/49) matching into adult or pediatric neurology.

### OA Exit Survey

#### Demographics

Thirty-three OAs completed and graduated from the program between 2012 and 2021 and thus were eligible to take the exit survey. Of those, 24 fully responded (73%).

#### Level 1, Reaction

Most OAs (n = 21, 88%) stated that they would recommend the program to medical students. Qualitative analysis of free-text responses to the question “Please share what you feel are the program's strengths” highlighted themes of exposure to academic MedEd and educators, opportunities for career development, research and teaching skills, and mentorship (Table 2).

**Table 2:** Representative Quotations From OA Exit Survey

Themes	Representative quotations
Please share what you feel are the program's strengths	
Exposure to academic medical education	Through the program, I gained much greater knowledge and much more experience about the running of a clerkship, designing effective medical education programs, clerkship and medical education leadership, and designing a research project in medical education
Career development	Exposure to the many different duties of a physician educator. Focus on improving teaching skills with feedback from course directors. Opportunity to design and implement med-ed research project
Exposure to clerkship administration	One of the greatest strengths of the program is the Neurology clerkship leadership team and the exceptional faculty mentors that students have in the OA program. We can learn so much just by watching how the team oversees the Neurology core clerkship, responds to feedback about the clerkship, and has adapted the clerkship in response to the pandemic
Teaching skills	The opportunity to grow as an educator while still in medical school was amazing. […]The responsibility afforded to us as OAs both in terms of the grading process and in leading discussions and review sessions was outstanding
Design and implementation of a scholarly project	I believe it's invaluable to have the opportunity to take on a research project in education (at any stage in its development) and be able to present it/have a product for the IEE. It's great as a learning experience and for students to gain additional exposure to medical education research and innovation projects
Mentorship	The one-on-one mentorship with the program leadership is by far the biggest strength. I learned so much about medical education, research, and my developing my own professional identity from all of the program leadership. They were always available and willing to help with anything: OA-related or not

Abbreviations: CD = clerkship director; IEE = Institute for Excellence in Education; OA = Osler Apprenticeship.

#### Level 2, Learning

Most of the OAs agreed that the experience enhanced their understanding of the roles and responsibilities of an academic educator (n = 24, 100%), improved their communication skills as an educator (n = 24, 100%), improved their leadership skills (n = 22, 92%), enhanced their skills and understanding of educational research design and scholarship (n = 22, 92%), and increased their comfort and confidence with being a resident educator (n = 22, 92%) (Table 3). Analysis of scholarly output during the program between 2012 and 2021 shows that 94% (31/33) of OAs submitted an abstract to a local/national conference, most completed or published a manuscript or book chapter, and many had more than 1 publication and were first authors (Table 4).

**Table 3:** Likert Responses to OA Exit and Alumni Surveys

OA exit survey question	Answered 4 or more on corresponding 5-point Likert scale
Has your experience as an OA influenced your academic practices and behaviors?^a^	22 (92%)
The OA experience^a^	
Enhanced my understanding of the roles and responsibilities of an academic educator	24 (100%)
Improved my communication skills as an educator	24 (100%)
Improved my leadership skills	22 (92%)
Enhanced my skills and understanding of educational research design and scholarship	22 (92%)
Increased my comfort and confidence with being a resident educator	22 (92%)
Alumni survey question	
To what extent do you agree or disagree?^b^	
My experience as an OA influenced and/or will influence my academic practices and behaviors	14 (78%)
After participating in the OA program, how confident or unconfident did you feel participating in: during residency and/or fellowship?^c^	
Medical education activities	15 (83%)
Medical education leadership	12 (67%)
Medical education research and scholarship	15 (83%)
How confident or unconfident do you feel: within medical education?^c^	
As a role model	13 (72%)
As a mentor	12 (67%)
As a teacher	16 (89%)
When conducting scholarship	14 (78%)
How often do or did you utilize what you learned from the OA program during residency and/or fellowship?^d^	5 (27%)
To what extent do you believe participating in the OA program resulted in you becoming a more favorable candidate during the residency and/or fellowship match process?^e^	12 (67%)

Abbreviation: OA = Osler Apprenticeship.

a 5-point scale of strongly disagree to strongly agree.

b 5-point scale of strongly disagree to strongly agree.

c 5-point scale of very unconfident to very confident.

d 5-point scale of never to always.

e 5-point scale of not at all to extremely.

**Table 4:** Scholarly Output and Awards

Scholarly output	%
Submitted abstract to local/national conference	94% (31/33)
Poster presentation	48% (16/33)
Oral presentation	51% (17/33)
Manuscript or book chapter completed	67% (22/33)
Manuscript or book chapter published	58% (19/33)
First author in published manuscript or book chapter	42% (8/19)
More than 1 publication^a^	58% (19/33)
Awards	
Earned by Osler Apprentices	33% (11/33)
AAN excellence in teaching award	45% (5/11)
IEE outstanding abstract	27% (3/11)
Other^b^	27% (3/11)
Earned by alumni^c^	39% (7/18)

Abbreviations: AAN = American Academy of Neurology; IEE = Institute for Excellence in Education; OA = Osler Apprenticeship.

a Including abstracts, posters, presentations, or manuscripts/book chapters.

b Including AAN minority travel scholarship, American Medical Association health system science challenge award, and medical student research in neurology award.

c Refers to awards, award nominations, educational recognition, or selection earned by alumni.

#### Level 3, Behavior

Most OAs (n = 22, 92%) agreed that their experience influenced their academic practices and behaviors. Based on OA program tracking data, one-third of students (n = 11, 33%) received an educational award or recognition (required drivers) while participating in the program (Table 4).^39^ Two-thirds (n = 16, 67%) of OAs reported plans to continue collaboration with directors.

### Alumni Survey

#### Demographics

Eighteen of 29 alumni (62%) fully responded to the alumni survey (1 survey was incomplete and excluded), and 9 of 29 (31%) uploaded a CV. All alumni (n = 18, 100%) stated that they currently work in a teaching hospital. Of those, 9 alumni (50%) are residents, 4 (22%) are fellows, and 5 (28%) are attendings. Fourteen alumni (78%) specialized in adult or pediatric neurology. Most alumni (n = 11, 61%) had not been involved in MedEd roles, leadership, or scholarship before the OA program. Of those who were involved in MedEd before the program, none were involved in formal MedEd training or programs.

#### Level 1, Reaction

All alumni (n = 18, 100%) stated that they would recommend the program to medical students, and nearly all (n = 17, 94%) felt that participation in the program strengthened their candidacy in residency or fellowship match. OA alumni highlighted many strengths of the program, including introduction to MedEd scholarship; opportunities to teach, educate, and lead; and connections with mentors (Table 5). Eight alumni CVs (89%) still included the OA program.

**Table 5:** Representative Quotations From the Alumni Survey

Themes	Representative quotations
What do you view as the strengths of the OA program?	
An introduction to medical education scholarship	I thought the opportunity to engage in curriculum change was a unique one. I also appreciated the exposure to medical education research
Good opportunities for teaching, medical education, and leadership	It gave me the opportunity to participate in medical education in a sustained and multifaceted manner, from peer teaching to curriculum design to scholarship and more
Connections with mentors, faculty, and leaders	Good mentorship from the leaders of the program, structured Med Ed research project, well organized, good opportunities for networking

Abbreviations: OA = Osler Apprenticeship.

#### Level 2, Learning

After participating in the OA program, most alumni felt confident participating in MedEd activities (n = 15, 83%), research and scholarship (n = 15, 83%), and leadership (n = 12, 67%) during residency and/or fellowship. Most alumni felt confident as a role model (n = 13, 72%), mentor (n = 12, 67%), and teacher (n = 16, 89%) within MedEd. Fourteen (78%) felt confident conducting MedEd scholarship (Table 3).

#### Level 3, Behavior

OA alumni (n = 14, 78%) agreed that their experience as an Osler Apprentice influenced their academic practices and behaviors. Half of all alumni (n = 9) reported using what they learned from the program about half of the time or more during residency and/or fellowship. Seven alumni (39%) said the availability of a MedEd curriculum/track was an important factor when applying to residency programs or creating a rank list. Most alumni (n = 13, 72%) went to a residency with a formal MedEd curriculum and/or track; of those, 8 (62%) participated. After medical school, one-third of alumni (n = 6, 33%) participated in MedEd workshops, courses, or certificate/degree programs to advance their career and/or leadership development; 14 (78%) were involved in MedEd-related scholarly work; and 16 (89%) were involved in educational activities such as teaching; developing courses, teaching materials, or curricular plans; or facilitating curricular change. This roughly corresponded to the CVs (comparison of CV and survey results provided in eTable 1). Seven alumni (39%) reported receiving an educational recognition, selection, award, or award nomination after medical school (Table 4).

#### Level 4, Results

When given a selection of leadership positions (such as residency/clerkship director [CD] or chief of education), 2 alumni (11%) indicated that they currently have a leadership position within MedEd. Nearly half (n = 8, 44%) aspire to have a leadership position within MedEd, with the greatest interest in CD (5 selections, 63%), fellowship director (4 selections, 50%), and residency assistant/associate program director (4 selections, 50%). CV analysis revealed that 4 of 8 alumni (50%) who were at or above neurology PGY-3 level in training at the time of CV analysis held or were holding the position of chief neurology resident. Ten alumni (56%) have an educational role (eTable 2).

### OA Preceptor Survey

Of 7 OA preceptors, 6 (86%) fully responded. Four preceptors were from the OA in Neurology, and 2 were from the OA in Pediatrics. The OA in Pediatrics was developed as a result of the OA in Neurology, and both groups were surveyed to gather a broader preceptor feedback. Preceptors most frequently indicated that they participated in the OA to enhance their skills as a mentor (n = 5, 83%), conduct educational research (n = 5, 83%), and play an important role in the lives and medical careers of students (n = 4, 67%). When exploring preceptor motivators of participation, preceptors primarily indicated participation due to career development or personal reasons (18/30 selections, 60%) rather than exclusively altruistic reasons (7/30 selections, 23%) such as connecting with students or making a difference in MedEd. All preceptors reported having at least 2 publications (including presentations, abstracts, or posters) through participation in the program. Three preceptors (50%) published more than 8 works during the course of their involvement. This was in line with the PubMed search analysis, which revealed that 4 of 6 preceptors (67%) had at least 1 publication. Three preceptors (50%) stated that their OA involvement was part of their promotion package. Half of the preceptors (n = 3, 50%) reported receiving a teaching award or recognition because of participation in the program, including teaching, professor, and alumni awards and a clerkship innovation award from the American Academy of Neurology.

## Discussion and Lessons Learned

Previous studies have evaluated largely research-oriented programs and workshops that aim to expose medical students to a career in MedEd.^12-17^ This report describes a comprehensive and longitudinal MedEd curriculum for medical students, addressing a gap in the existing literature. We also explored learner and faculty development within this curriculum, focusing on the program's impact on participants and the motivations and benefits experienced by preceptors. Using the NWKM framework, we identified a significant impact and success of the OA at levels 1–3 and found that the program supported the careers of preceptors (academic faculty).^36,39^

### The Apprenticeship

The OA in Neurology aims to inspire and prepare students to pursue careers as medical educators, while also fostering productivity for faculty and the NCC, ensuring the program's long-term sustainability. The OA uses neurology as a pathway to enhance understanding of the roles of a medical educator through its key program components. The program provides students with mentorship through longitudinal apprenticeships with directors who serve as “coaches,” fostering student interest and productivity while guiding them through execution of a scholarly project. Bringing the apprenticeship model into the modern era, the CliftonStrengths workshop is used to help students identify and leverage their strengths as they move forward in the program.^40,41^ By understanding their strengths early, students can focus on their skill sets and collaborate more effectively, applying this knowledge while serving as leaders for NCC students. This allows students to act as mentors in a low-stakes environment where they can receive feedback and adjust their leadership styles. Through other structured experiential learning opportunities such as CD meetings, students gain insight into the multifaceted role of a medical educator.^42^ As the field of neurology seeks to expand its pool of educators, formal training and early exposure to all facets of MedEd are of necessity.

### Evaluation Using the NWKM

**Figure 2:**
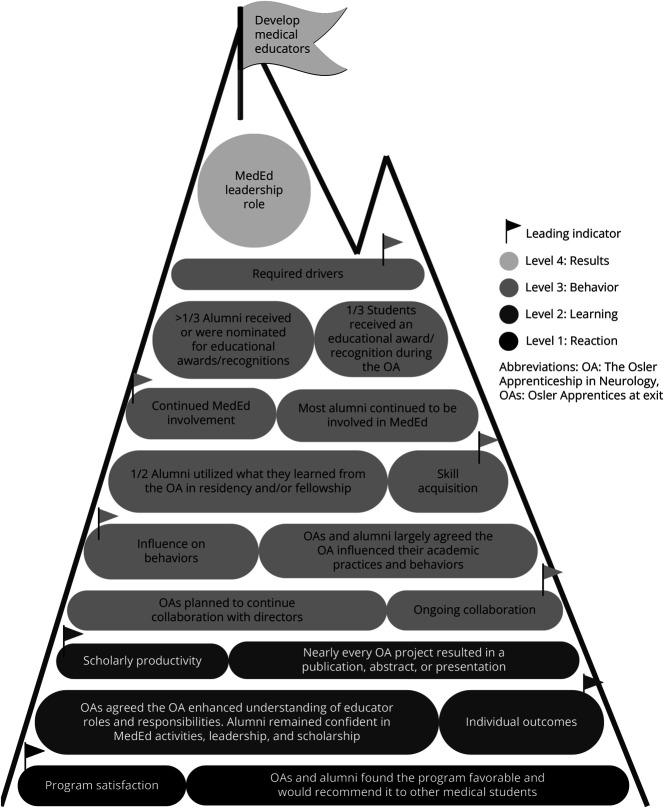
Leading Indicators

We used the NWKM to conduct an evaluation of the OA and identified substantial impact and success of the program at levels 1–3.^36,39^ When assessing level 4, which evaluates whether the desired outcome (OA serving as a pathway to introduce students to a career in MedEd to aid in the development of medical educators) occurs because of the learning event, we cannot definitively state whether our level 4 results, including most of the alumni having an active educational or leadership role, occurred as a direct result of the OA. However, leading indicators at levels 1–3 such as program satisfaction, OA scholarly productivity in MedEd, the OA's influence on behaviors, alumni involvement in MedEd, and required drivers (motivators, and in our case, awards/recognitions) suggest that behaviors are on track to achieve the desired results to aid in the development of medical educators. The NWKM highlights that when evaluating level 4, leading indicators can be viewed as targets, acting as flags on the path toward desired results at the summit, with each flag contributing to the level 4 result of the program. From our evaluation, leading indicators of the OA program are presented in Figure 2, highlighting progress toward our goal: the development of medical educators.

Notably, in our cohort of students, nearly a quarter were first authors of a publication in comparison with 13% in the general medical student population. Medical students who participate in research have been shown to be over 6 times as likely to pursue academic careers and over 3 times more likely to report interest in research involvement during their future careers.^34^ In addition, experiential opportunities, leadership, and mentorship, all of which are components of the OA, have been emphasized and correlated with greater recruitment of learners into academia.^8,21,34,43^ Along with the leading indicators mentioned above, this suggests that the OA is providing key elements needed to guide students on the path toward becoming medical educators.

### Success as a “Win-Win”

In 2019, the AAN created a workgroup to address educational funding in neurology, including identifying sources and obstacles for the funding of neurology educators. They highlighted that indirect methods for awarding and incentivizing education are needed.^44^ For preceptors, participation in the OA resulted in robust scholarly output, innovation, and teaching awards and aided in the academic promotion process. In an era of burnout and increasing physician workload, particularly in neurology, these additional incentives are necessary to recruit faculty to participate in teaching activities.^25-27,44^ Less than a quarter of OA preceptors stated that they participated in the program because of altruistic reasons such as connecting with students and playing an important role in their lives. Rather, most ascribed participation to career development and personal gains. An emerging body of scholarship on faculty vitality highlights efficiency as a key factor for faculty to be able to balance their activities.^22^ As the role of the neurology medical educator develops, educators will be expected to teach, collaborate with colleagues, keep up with advancements, and conduct meaningful scholarship, and the OA provides an integrated opportunity to achieve all the above.

### Room for Improvement and Limitations

The OA has evolved based on feedback. When analyzing areas for improvement suggested by participants and alumni, a common theme included greater structure and clearly defined projects and more teaching and formal didactic opportunities (eTable 3). Because didactic opportunities depend on departmental and clerkship needs, structured programming remains limited. In the future, the OA program could implement additional formal teaching opportunities.

Our study had limitations. The absence of demographic data limits the generalizability. Surveys were based on self-reported responses with retrospective questions, representing the possibility of recall bias, although CV analysis and literature searches helped mitigate this. It is also possible that not all publications were indexed in PubMed or that CVs were not fully updated. Free-response survey questions were used and prone to data subjectivity when analyzed. OA exit and alumni surveys were administered independently and were not matched to the same respondents. The alumni survey may have been interpreted differently among respondents, specifically when addressing involvements in MedEd activities, roles, and leadership. Only 2 alumni stated that they have a current leadership position within MedEd; however, many educational roles cited by alumni could be considered leadership positions (e.g., chief resident). Selection bias is also a factor because OAs may have pursued careers and roles in MedEd regardless of their participation in the program and applicants were limited to students who earned high pass or honors grades in the NCC. However, survey responses indicate that the OA program provided the tools and guidance for students to better serve as medical educators. Selection bias is relevant to preceptors as well who may have already had high interest and skills in MedEd, making them more likely to earn awards or recognition, regardless of their participation in the OA. Finally, our study lacked comparison groups, making it difficult to determine the program's direct impact on participants and preceptors and establish whether preceptors' scholarly activity was augmented through the program, rather than stemming from it. Future studies including a control group could validate our findings, particularly now that there are additional OA programs.

## Closing Remarks

Our evaluation suggests that the OA is making effective and impactful steps toward building a stronger pathway for medical educators while simultaneously supporting the careers of academic medical faculty, allowing for program sustainability. To cultivate and sustain future generations of neurology educators, we envision the OA serving as a prototype for the establishment of more formalized training programs in neurology MedEd. As a testament to its success, OA programs have also been implemented in the JHU SOM pediatrics, obstetrics and gynecology, internal medicine, emergency medicine, and surgery clerkships. The program has inspired similar programs at other institutions including Baylor, University of Michigan, and University of Maryland.^45^

The implementation of programs such as the OA represents just 1 milestone in the ongoing mission to advance neurology education at JHU SOM. Since its establishment in 1980, the NCC at JHU SOM has evolved to cultivate skilled educators. Key advancements include increasing full-time equivalent support for CDs and coordinators, expanding the CD team and roles, integrating HSS (including the OA) into the NCC curriculum, and formally recognizing faculty educators through the Osler Preceptors in Neurology Program.^31^ We hope that the OA can continue to help cultivate the next generation of medical educators within neurology and other medical specialties. However, sustained advancement in neurology education requires continuous and targeted improvements and collaboration at the departmental, clerkship, faculty, and student levels.

## Glossary

AAN: American Academy of Neurology
CD: clerkship director
CV: curriculum vitae
HSS: Health Systems Science
IEE: Institute for Excellence in Education
JHU: Johns Hopkins University
MedEd: medical education
NCC: Neurology Core Clerkship
NWKM: New World Kirkpatrick Model
OA: Osler Apprenticeship
SOM: School of Medicine
